# Efficacy and safety of different regimens of neoadjuvant therapy in patients with hormone receptor-positive, her2-negative breast cancer: a network meta-analysis

**DOI:** 10.3389/fimmu.2024.1420214

**Published:** 2024-08-23

**Authors:** Yongxiao Wu, Shibo Huang, Yanlin Wei, Miaoyan Huang, Chunyan Li, Weiming Liang, Tian Qin

**Affiliations:** The First Affiliated Hospital of Guangxi University of Science and Technology, Guangxi University of Science and Technology, Liuzhou, Guangxi, China

**Keywords:** breast cancer, olaparib, nivolumab, neoadjuvant therapy, pathological complete response, HER2 negative, network meta-analysis

## Abstract

**Introduction:**

The objective of this systematic review and network meta-analysis (NMA) is to assess the effectiveness and safety of various neoadjuvant treatment protocols in individuals diagnosed with hormone receptor-positive, her2 negative(HR+/HER2-) breast cancer.

**Materials and methods:**

A systematic search was conducted in four databases (Medline, Embase, Web of Science, and CENTRAL) from the inception of the databases to January 16, 2024, to identify randomized controlled trials (RCTs) to various neoadjuvant therapy options in patients diagnosed with hormone receptor-positive, HER2-negative breast cancer. A network meta-analysis was conducted to evaluate pathological complete response (pCR).

**Results:**

There were 17 randomized controlled trials (RCTs) included in the analysis. These trials examined 16 different treatment regimens and involved a total of 5752 participants. The analysis revealed that the six most effective neoadjuvant treatment regimens for HR+/HER2- breast cancer were: CT(A)+olaparib (82.5%), CT(A)+nivolumab (76.5%), Com (74.9%), CT (72.1%), Mono+eribulin (72.0%), and CT(A)+pembrolizumab (70.4%).Paired meta-analysis for pathological complete response (pCR) found no statistically significant distinction between treatment regimens that included both anthracycline and immunosuppressants and regimens that relied solely on anthracycline chemotherapy(OR:1.14, 95%ci 0.79-1.64, I^2^ = 71%, P=0.50). Similarly, there was no significant difference between platinum-based chemotherapy and anthracycline-basedchemotherapy(OR:1.37, 95%ci 0.53- 3.56, I^2^ = 11%, P=0.52). With regards to safety, adverse effects of grade 3-5 were observed, which included haematological toxicity, gastrointestinal reactions, skin and mucous membrane reactions, neuropathy, hepatotoxicity, and cardiac disorders.

**Conclusions:**

The CT(A)+Olaparib and CT(A)+nivolumab groups demonstrated superior efficacy in neoadjuvant therapy for HR+/HER2- breast cancer. Furthermore, it is crucial to focus on effectively managing the adverse effects of the treatment plan to enhance patient’s ability to tolerate it. Given the constraints of the current research, additional well-executed and suitable RCTs are necessary to validate the findings of this investigation. Although pCR is valuable in assessing the effect of neoadjuvant therapy in some cases, prognostic prediction and efficacy assessment in patients with HR+/HER2- breast cancer should be based on a combination of broader clinical and biological characteristics.

**Systematic review registration:**

PROSPERO https://www.crd.york.ac.uk/prospero/display_record.php?ID=CRD42024534539, CRD42024501740.

## 1 Introduction

Breast cancer is a prevalent form of cancerous tumors in women globally, and its occurrence is progressively rising annually. Breast cancer has emerged as a prominent contributor to the mortality rate of women globally. Worldwide, breast cancer constitutes around 30% of all female malignancies, and has a death and morbidity rate of 15% (1). Breast cancer incidence is influenced by a range of factors, such as genetic predisposition, lifestyle choices, hormone levels, and environmental exposures. Although there have been notable advancements in the early detection and treatment of breast cancer in recent times, there remains a continued risk of recurrence or metastasis for many individuals (2). Breast cancer is categorized into three primary subtypes based on the presence of hormone receptors (ER and PR) and HER2 (ERBB2) status: luminal ER-positive and PR-positive, which can be further divided into luminal A and B; HER2-positive; and triple-negative breast cancer (TNBC) (3).

Neoadjuvant therapy is a crucial clinical strategy used in the treatment of breast cancer. It is administered before the primary treatment to improve therapeutic outcomes. The primary goals of neoadjuvant therapy are to decrease tumor size, enhance the possibility of surgical removal, lower the chances of metastasis and recurrence, and improve patient survival rates (4). Neoadjuvant therapy differs from typical preoperative treatments by placing greater emphasis on considering the unique characteristics of each patient and the biology of their tumor. This approach enables the development of a more precise and personalized treatment strategy for each individual. Following neoadjuvant chemotherapy, patients experience pathological complete response (pCR) and may exhibit improved survival results (5, 6).The selection of neoadjuvant therapy for breast cancer is determined by the patient’s physiological condition, such as age, menopausal status, underlying diseases, and the pathological characteristics of the cancer, including tumor size, lymph node involvement, hormone receptor status, HER2 expression, and Ki-67 expression (7).

Neoadjuvant trials investigating the use of ‘targeted’ therapy and tumor subtype design have provided evidence supporting the predictive value of pathological complete response (pCR) in HER2+ and TN EBC. As a result, neoadjuvant chemotherapy (NACT) combined with targeted therapy in HER2+ tumors has become the recommended treatment for stage II-III HER2+ and TN EBC (8). Nevertheless, there are lingering debates and difficulties in implementing neoadjuvant therapy for breast cancer, particularly in patients with HR+/HER2 negative breast cancer. The selection of treatment plan for various subtypes of breast cancer, particularly in HER-2 negative, hormone receptor positive patients, is still unknown (9). Another issue that needs to be addressed is the potential for toxic responses and side effects when neoadjuvant therapy is used in clinical settings. Finding a balance between therapeutic efficacy and safety is also challenging. Furthermore, there are still certain limits to the deployment of personalized treatment plans in neoadjuvant breast cancer treatment. One of the main goals of current research is to optimize treatment protocols by making greater use of genetic testing technology and molecular markers.

Network meta-analysis is a method that allows for the interpretation of randomized evidence from a network of trials. It has the ability to rate several treatments, surpassing the traditional approach of only comparing treatments directly (10–12). Network meta-analyses have become popular due to the growing complexity of analyzing clinical guideline databases and decision-making processes by policymakers. In this study, we conducted a comprehensive evaluation of the effectiveness and safety of neoadjuvant therapy for hormone receptor-positive, Her2-negative breast cancer. Our assessment involved a rigorous comparison of multiple randomized clinical trials, both directly and indirectly.

## 2 Materials and methods

### 2.1 Search strategy

This meta-analysis adhered to the 2020 requirements of the Preferred Reporting Project for Systematic Review and Meta-Analysis (PRISMA). The study has been registered at PROSPERO with the registration number CRD42024501740. A systematic search was conducted in four databases, namely PubMed, Embase, Web of Science, and the Cochrane Library, from literature published until January 16, 2024. The search strategy followed the PICOS principle and involved a combination of MeSH terms and free-text words. The specific search strategy used was (“HER-2” AND “Breast Cancer” AND “Neoadjuvant therapy” AND “randomized controlled trial”). **Supplementary Table 1** provided a comprehensive overview of the search record.

### 2.2 Inclusion and exclusion criteria

Inclusion criteria were as follows: (1)patients diagnosed as hormone receptor-positive, her2-negative breast cancer; (2) two distinct groups of patients were administered varying neoadjuvant chemotherapy regimens; (3) pCR was reported; (4)study design was randomised controlled trial.

Exclusion criteria: (1)other types of articles, such as case reports, letters, reviews, meta-analyses, editorials, animal studies and protocols; (2) not RCTs; (3) pCR was not reported; (4) Reduplicate cohort of patients. (5)Studies on other types of breast cancer.

### 2.3 Selection of studies

The literature selection procedure, which included the elimination of duplicate entries, was carried out using EndNote (Version 20; Clarivate Analytics).The initial search was conducted by two autonomous reviewers. The redundant items were removed, and the titles and abstracts were evaluated to determine their relevance. Subsequently, each study was classified as either included or omitted. We settled the matter by coming to an agreement through mutual accord. In the event that the parties involved are unable to come to a mutual agreement, a third reviewer assumes the function of a mediator.

### 2.4 Data extraction

Data extraction was conducted by two researchers working independently, and any differences that emerged were resolved by a third researcher. The recorded information encompassed the research’s details such as the name of the first author, the year of publication, the NCT study design, the trial phase, the recruitment period, the treatment administered, the sample size, the age of the patients, the primary result (pCR), and any adverse events (AEs) observed.

### 2.5 Risk of bias assessment

Two independent reviewers evaluated the potential for bias in the trials included by utilizing the Cochrane Risk of Bias tool. This assessment focused on various domains, including random sequence generation, allocation concealment, blinding of participants and personnel, blinding of outcome assessment, incomplete outcome data, selective reporting, and other sources of bias. In the event of any inconsistencies, the contentious findings were resolved by collective deliberation.

### 2.6 Statistical analysis

The odds ratio (OR) was utilized to calculate the combined effect sizes. Heterogeneity in pairwise meta-analysis was assessed using the Cochrane Q statistic and the I^2^ test. A meta-analysis was conducted using either a random-effects model or a fixed-effects model, depending on the presence of statistical heterogeneity. Review Manager version was used to conduct pairwise meta-analyses. All P-values were two-sided, and the difference in P value less than 0.05 was statistically significant (13). The congruity between direct and indirect evidence was confirmed using node splitting analysis. If no significant contradiction was identified, a consistency model was employed to examine the relative effects of the interventions. Alternatively, an inconsistency model was utilized. We utilized the “network” features of Stata (v15.0) software to arrange conversations and do data analysis (14). The network meta-analyses yield results in the form of odds ratios (OR) and their related 95% CI. Probabilities were computed to rank each treatment and determine their respective ranks. When evaluating the effectiveness of a drug, we use the surface under the cumulative ranking curve (SUCRA) value. A higher SUCRA value indicates that the drug performs better.

## 3 Results

### 3.1 Search results

**Figure 1:**
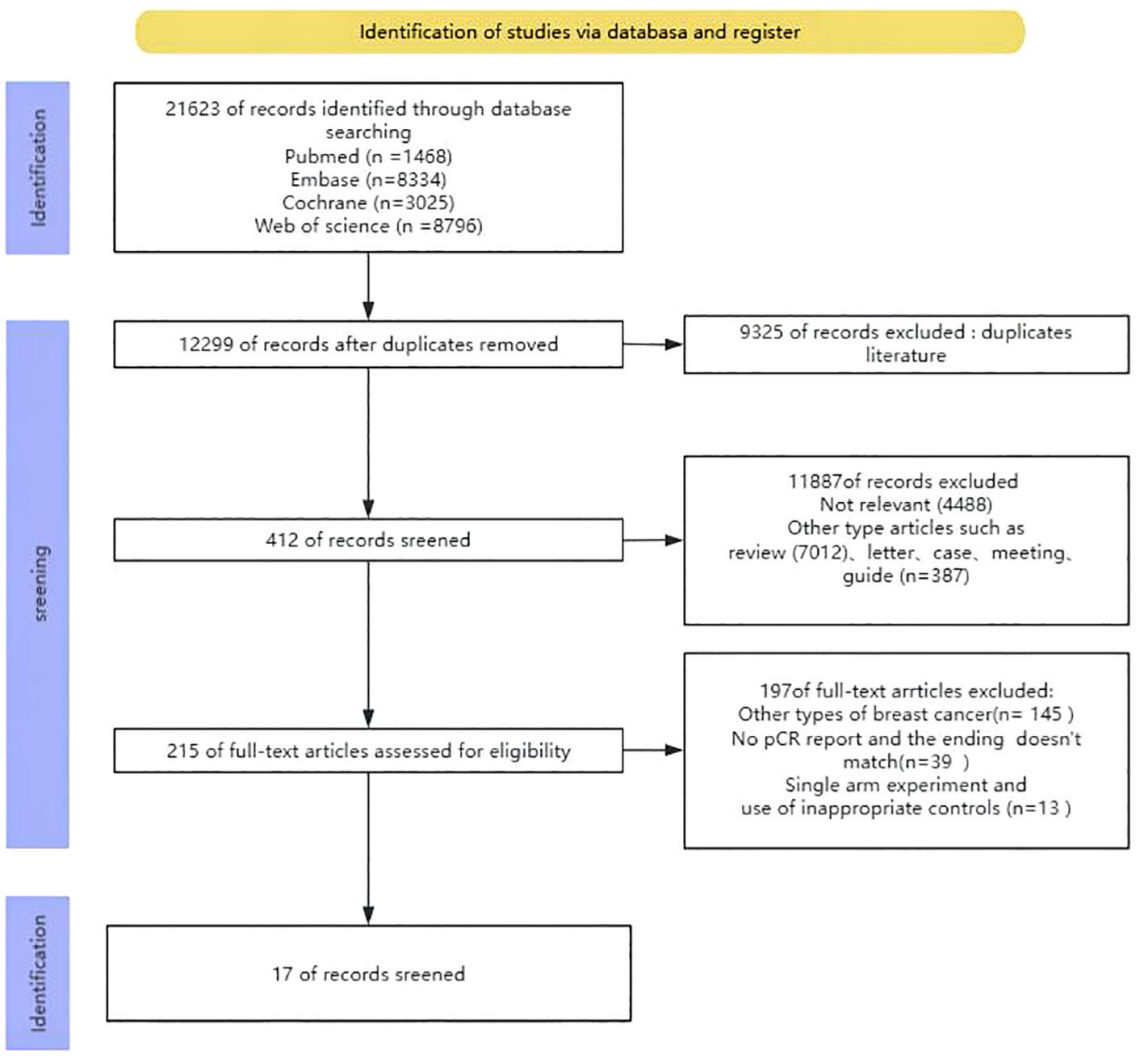
Flow chart of literature search strategies.

Upon conducting the initial search, a grand total of 21623 publications were discovered. Nevertheless, after eliminating redundant research, the total number of instances was reduced to 12299. After assessing the titles and abstracts, a grand number of 11887 papers were excluded from further consideration. In the end, a grand total of 215 articles were available for a thorough analysis of their entire content. Out of the 215 studies, 145 records focused on different forms of breast cancer, 39 entries did not provide information on the main outcome measure pCR, and 13 records were removed because they were single-arm studies and utilized unsuitable controls. In the end, only 17 studies were selected for analysis. The process of selecting and incorporating the literature was illustrated in **Figure 1**.

### 3.2 Basic characteristics of the included literature

**Figure 2:**
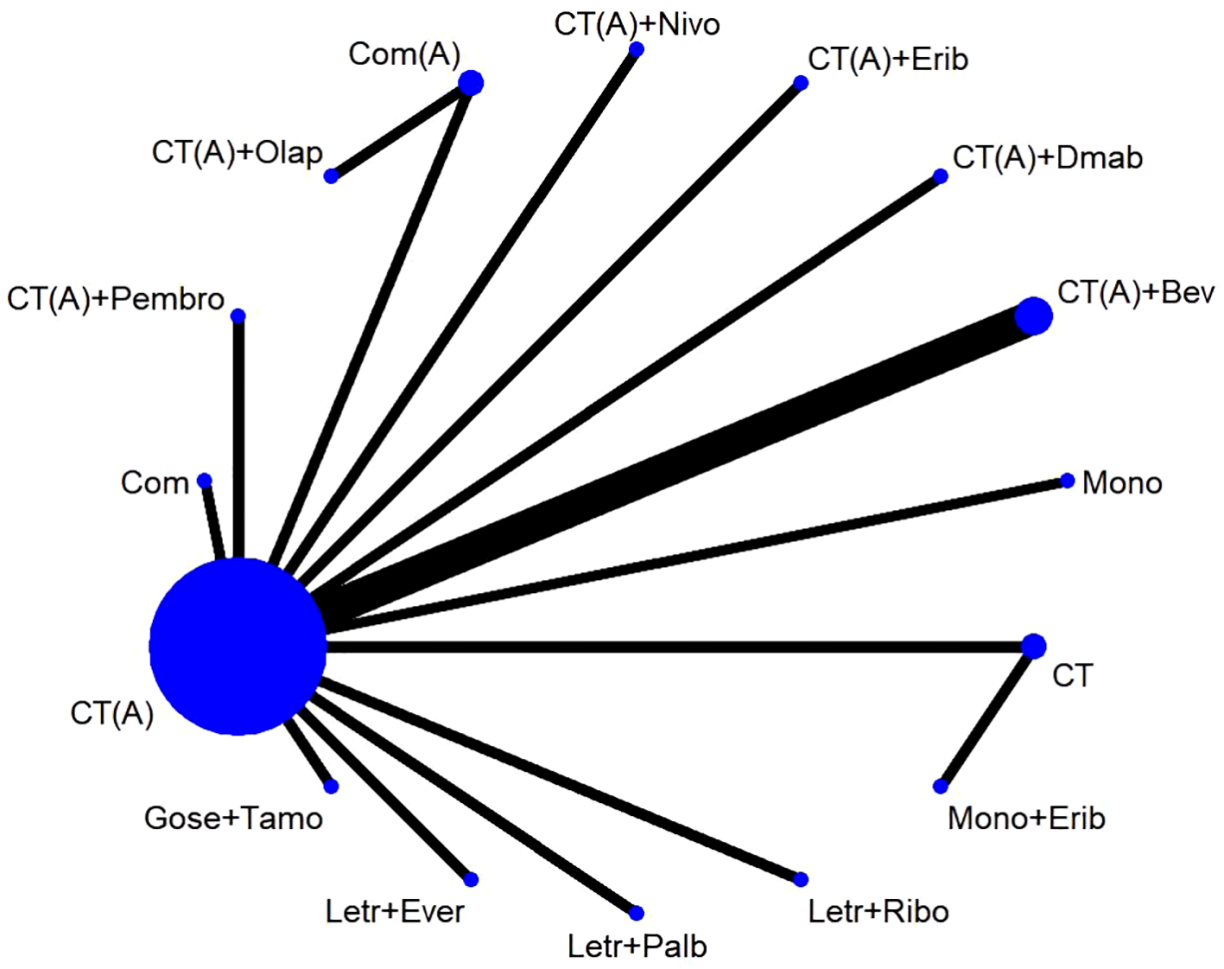
Network diagram.

The study included a total of 17 publications (15–31), all of which were two-arm randomized controlled trials (RCTs). Therefore, the original literature had 34 intervention arms, which were categorized into 16 treatment regimens based on the study design, as outlined below: (1) CT, chemotherapy alone (combination of 2 or more chemotherapeutic agents without anthracycline and platinum); (2) CT(A), anthracycline-containing chemotherapy; (3) CT(A) + Bev, anthracycline-containing chemotherapy + bevacizumab; (4) CT(A) + Pembro, anthracycline-containing chemotherapy + pembrolizumab; (5) CT(A)+Erib, anthracycline-containing chemotherapy + eribulin; (6) CT(A)+Olap, anthracycline-containing chemotherapy + olaparib; (7) CT(A)+Dmab, anthracycline-containing chemotherapy + denosumab; (8) CT(A)+Nivo, anthracycline-containing chemotherapy + Nivolumab; (9) Com, platinum-containing chemotherapy; (10) Com(A), platinum- and anthracycline-containing chemotherapy; (11) Letr+Ever, letrozole + everolimus; (12) Gose+Tamo, goserelin + tamoxifen; (13) Letr+Palb, letrozole + pepcidil; (14) Letr+Ribo, letrozole + ribociclib; (15) Mono, single-agent chemotherapy (one chemotherapeutic agent); (16) Mono+Erib, single-agent chemotherapy + eribulin. Out of the 17 randomized controlled trial (RCT) publications, the major outcome metric of pathological complete response (pCR) was reported (**Figure 2**).The study was balanced across groups and comparable at baseline, as detailed in the Basic Characteristics Information Sheet (**Table 1**).

**Table 1:** Characteristics of included studies and patients.

Study	Clinical trial	Study design	Recruitment time	regimens	Renamed treatment	patients no.	patient age	Efficacy endpoint
Earl 2015 (18)	NCT01093235	phase 3, randomised, open-label	2009.05-2013.01	Docetaxel + fluorouracil + epirubicin + cyclophosphamide	CT(A)	401	>=18	pCR AEs
				Bevacizumab + docetaxel + fluorouracil + epirubicin + cyclophosphamide	CT(A) + Bev	399	>=18	
Kim 2020 (23)	NCT01622361	Phase 3,randomised, open-label	2012.07-2017.05	Adriamycin + cyclophosphamide + docetaxel	CT(A)	87	42.5±5.6	pCR
				Goserelin + tamoxifen	Gose + Tamo	87	41.5±5.8	
Cottu 2018 (20)	NCT02400567	phase 2, randomised,parallel	2015.02-2016.11	letrozole + palbociclib	Letr + Palb	53	65(49-78)	pCR
				Docetaxel + fluorouracil + epirubicin + cyclophosphamide	CT(A)	53	62(48-80)	
Abraham 2015 (17)	NCT01705691	phase 2,randomized	2013.01-2013.08	Doxorubicin + cyclophosphamide + paclitaxel	CT(A)	19	48(34–67)	pCR、 AEs
				Eribulin + doxorubicin + cyclophosphamide	CT(A) + Erib	30	50(28–70)	
Yardley 2019 (21)	NCT01527487	phase 2, randomized, open-label	2012.07-2014.03	Eribulin + cyclophosphamide	Mono + Erib	54	53(23 -77)	pCR、 AEs
				Docetaxel + cyclophosphamide	CT	22	51(38 -73)	
Ando 2014 (16)	NA	phase 2, randomized, non-blinded	2010.03-2011.09	carboplatin + paclitaxel + fluorouracil + epirubicin + cyclophosphamide	Com(A)	88	47(30–69)	pCR、 AEs
				Paclitaxel + fluorouracil + epirubicin + cyclophosphamide	CT(A)	91	47(30–70)	
Ishiguro 2020 (22)	UMIN000003283	randomized	2010.01-2011.09	Docetaxel + cyclophosphamide + fluorouracil + epirubicin + cyclophosphamide	CT(A)	128	50(26–69)	pCR、 AEs
				Docetaxel + cyclophosphamide	CT	65	50(30–68)	
Nahleh 2016 (19)	NCT00856492	phase 2, randomized, open-label	2010.05-2012.09	Bevacizumab + Nab-paclitaxel + doxorubicin + cyclophosphamide	CT(A) + Bev	99	51.7(22–71)	pCR、 AEs
				Nab-paclitaxel + doxorubicin + cyclophosphamide	CT(A)	118	51.3(31–75 )	
Tung 2020 (25)	NCT01670500	phase 2, randomized	2012.01- 2019.01	Cisplatin	Mono	60	40±9	pCR、 AEs
				Doxorubicin + cyclophosphamide	CT(A)	58	44±10	
Yang 2022 (29)	ACTRN12613000206729	randomized	2012.09-2018.12	Docetaxel + capecitabine	Com	54	52(22-79)	pCR
				Docetaxel + epirubicin	CT(A)	59	52(22-79)	
Fasching 2020	NCT04436744	randomized	2020.09-2021.11	Paclitaxel + olaparib	CT(A) + Olap	112	63.1 (7.9)	pCR AEs
				Paclitaxel + carboplatinum	Com(A)	109	62.4 (9.3)	
Cardoso 2017	NCT03725059	phase 3, randomised, open-label	2018.10-2023.12	Pembrolizumab + paclitaxel + doxorubicin or epirubicin + cyclophosphamide	CT(A) + Pembeo	635	33.2 (9.7-51.8)	pCR AEs
				paclitaxel + doxorubicin or epirubicin + cyclophosphamide	CT(A)	643	33.2 (9.7-51.8)	
Prat 2019	NCT03248427	phase 2, randomized, open-label	2017.07-2018.12	Ribociclib + letrozole	Letr + Ribo	52	63(56.5–70.3)	pCR
				Doxorubicin + cyclophosphamide + paclitaxel	CT(A)	54	64(58.3–71.8)	
Blohmer 2022 (28)	NCT02682693	phase 2, randomized, open-label	2017.02-2019.03	Denosumab + nab-paclitaxel + epirubicin/cyclophosphamide	CT(A) + Dmab	153	NA	pCR AEs
				nab-paclitaxel + epirubicin/cyclophosphamide	CT(A)	157	NA	
Wu 2021 (27)	NCT02742051.	randomized	NA	Everolimus + letrozole	Letr + Ever	20	60.0 (54-70)	pCR AEs
				Fluorouracil + epirubicin + cyclophosphamide	CT(A)	20	56.5 (51-66)	
Minckwitz 2012	NCT00567554	randomized	NA	Epirubicin + cyclophosphamide + docetaxel	CT(A)	629	48 (24–78)	pCR AEs
				Epirubicin + cyclophosphamide + docetaxel + bevacizumab	CT(A) + Bev	633	49 (21–75)	
Loi 2024	NCT04109066	phase 3, randomized, open-label	2019.09-2024.03	Nivolumab + paclitaxel + anthracycline + cyclophosphamide	CT(A) + Nivo	253	NA	pCR AEs
				Paclitaxel + anthracycline + cyclophosphamide	CT(A)	257	NA	

### 3.3 Risk of bias

**Figure 3:**
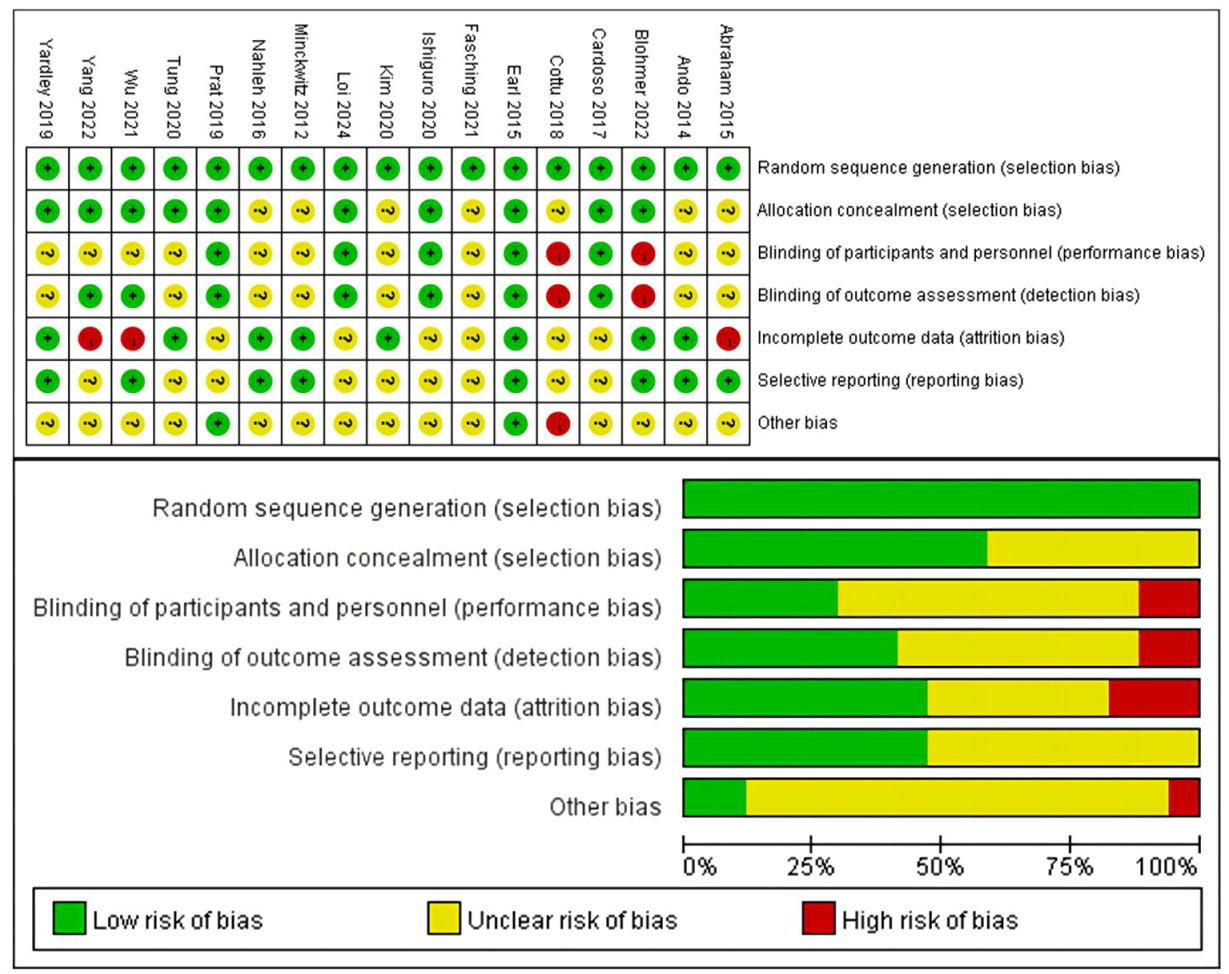
Risk of bias assessment diagram.

**Figure 3** provided a summary of the risk of bias assessment results. Among the 17 studies, an adequate randomized sequence was generated in 17 studies, appropriate allocation concealment was reported in 10 studies, the blinding of participants was clear in 5 studies, the blinding of outcome assessors was reported in 7 studies, outcome data were complete in 8 studies, 8 studies had no selective reporting, and 2 studies had no other bias. **Figure 3** displays the specific information regarding the risk of bias in the literature.

### 3.4 Paired meta-analysis for pCR

**Figure 4:**
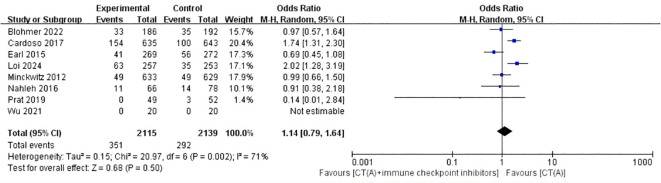
Forest plot of the meta-analysis for pCR [CT(A)+Immune checkpoint inhibitors vs CT(A)].

**Figure 5:**
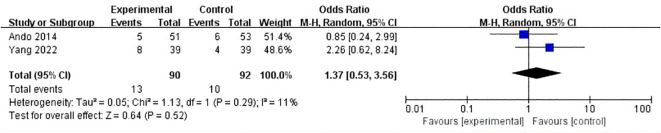
Forest plot of the meta-analysis for pCR [Com vs CT(A)].

Eight studies conducted a comparison between regimens consisting of anthracycline plus immunosuppressants and regimens based on anthracycline chemotherapy. The meta-analysis of these studies showed that there was no statistically significant difference between the two types of regimens (OR:1.14, 95%ci 0.79-1.64, I^2^ = 71%, P=0.50) (**Figure 4**). Two studies did a comparative analysis of platinum-based chemotherapy and anthracycline-based chemotherapy. The meta-analysis of these studies revealed that there was no statistically significant disparity between the two types of regimens (OR:1.37, 95%ci 0.53- 3.56, I^2^=11%, P=0.52) (**Figure 5**).

### 3.5 Network meta-analysis for pCR

**Figure 6:**
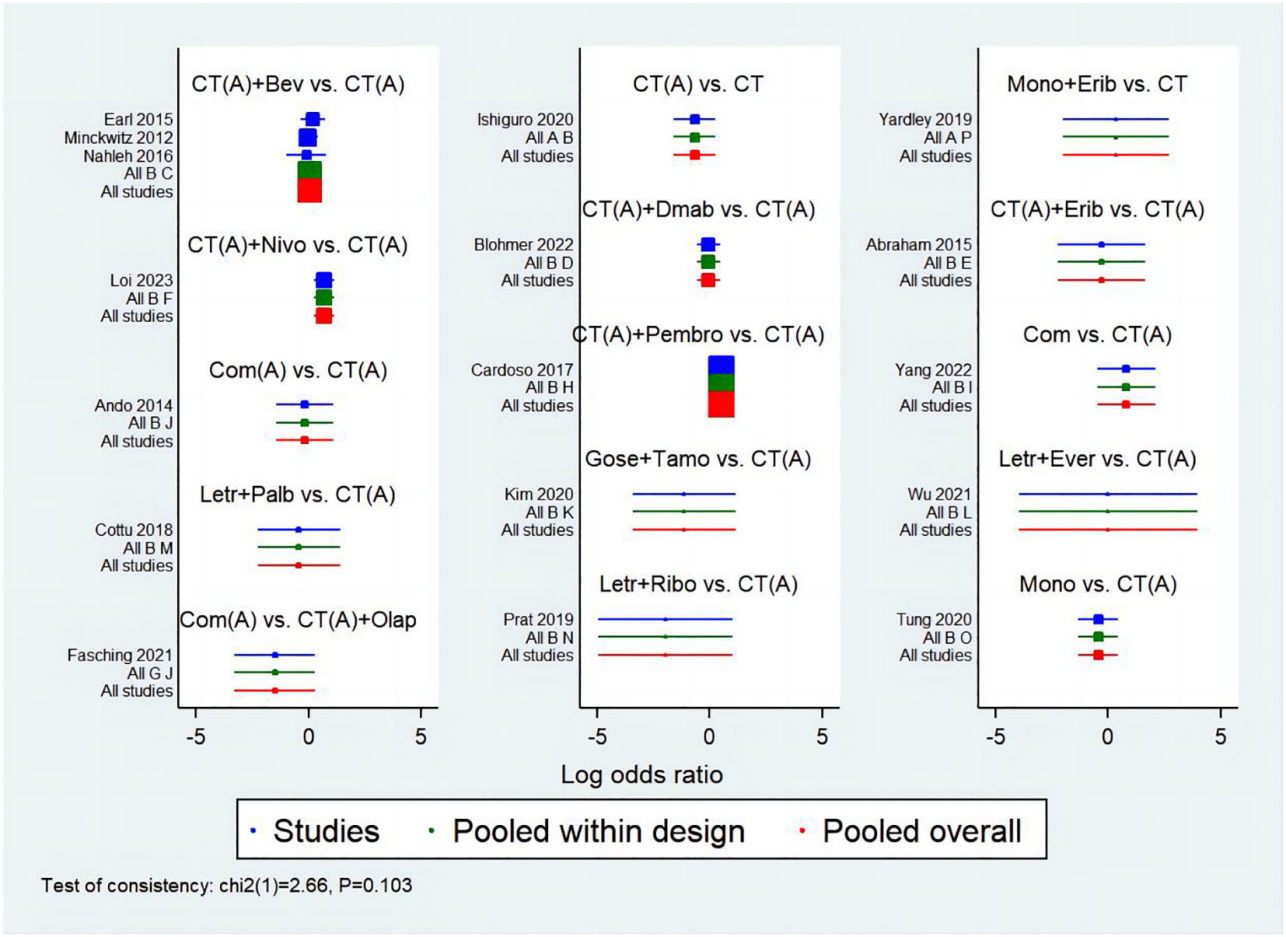
Consistency and inconsistency analysis charts.

**Figure 7:**
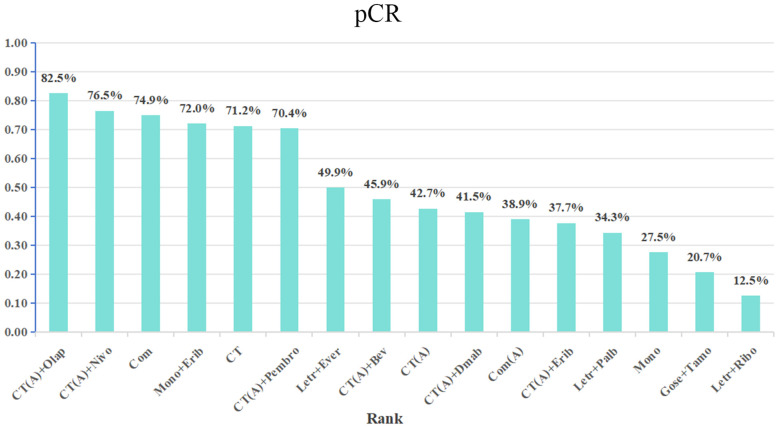
SUCRA Sorting Chart for pCR.

The network meta-analysis incorporated a total of 17 randomized controlled trials (RCTs) to evaluate the consistency and inconsistency models. The global inconsistency test yielded a P-value of 0.103 (**Figure 6**), showing a high level of consistency. There was no significant difference observed between direct and indirect comparisons, suggesting that the reticulated meta-analysis was reliable.According to the data presented in **Table 2**, the CT (A) regimen had a lower rate of pCR compared to the CT (A) + Pembro regimen (OR: 0.64, 95% CI 0.51-0.80) and the CT (A) + Nivo regimen (OR: 0.49, 95% CI 0.31-0.78). There were no significant differences observed in the direct comparisons between the other treatments. The results of indirect comparisons indicate that the Moon regimen(OR: 0.37, 95% CI 0.15-0.93) and CT (A)+Bev regimen(OR: 0.60, 95% CI 0.40-0.91) had significantly lower rates of pCR compared to the CT (A)+Pembro regimen. Compared to the CT(A)+Nivo regimen, the Moon regimen(OR: 0.32, 95% CI 0.12-0.86), CT (A)+Bev regimen(OR: 0.52, 95%CI 0.30-0.90), and CT(A)+Dmab regimen(OR: 0.48, 95% CI 0.24-0.96) exhibited a significantly lower pathological pCR rate.An analysis was conducted on the cumulative ranking of the 15 treatment regimens. The findings indicated that the treatment outcomes were arranged in order of superiority, ranging from the most favorable to the least favorable: CT(A)+Olap (82.5%), CT(A)+Nivo (76.5%), Com (74.9%), CT (72.1%), Mono+Erib (72.0%), CT(A)+Pembeo (70.4%), Letr+Ever (49.9%), CT(A)+Bev (45.9%), CT(A) (42.7%), CT(A)+Dmab (41.5%), Com(A) (38.9%), CT(A)+Erib (37.7%), Letr+Palb (34.3%), Mono (27.5%), Gose+tamo (20.7%), Letr+Ribo (12.5%) (**Figure 7**).

**Table 2:** League table for pCR.

Letr+Ribo														
0.44 (0.01- 18.87)	Gose+Tamo													
0.22 (0.01- 5.01)	0.51 (0.04- 5.84)	Mono												
0.22 (0.01- 7.28)	0.50 (0.03- 9.30)	0.98 (0.13- 7.51)	Letr+Palb											
0.19 (0.01- 6.80)	0.43 (0.02- 8.80)	0.86 (0.10- 7.35)	0.87 (0.06- 12.74)	CT(A)+Erib										
0.17 (0.01- 4.29)	0.38 (0.03- 5.17)	0.76 (0.16- 3.50)	0.77 (0.08- 7.07)	0.88 (0.09- 9.03)	Com(A)									
0.15 (0.01- 3.07)	0.34 (0.03- 3.50)	0.67 (0.24- 1.85)	0.68 (0.10- 4.54)	0.78 (0.10- 5.90)	0.88 (0.23- 3.43)	CT(A)+Dmab								
0.14 (0.01- 2.84)	0.33 (0.03- 3.19)	0.64 (0.27- 1.55)	0.65 (0.10- 4.08)	0.75 (0.11- 5.32)	0.85 (0.24- 2.99)	0.97 (0.57- 1.64)	CT(A)							
0.14 (0.01- 2.75)	0.31 (0.03- 3.10)	0.61 (0.24- 1.56)	0.62 (0.10- 3.99)	0.71 (0.10- 5.19)	0.81 (0.22- 2.95)	0.92 (0.50- 1.69)	0.95 (0.70- 1.29)	CT(A)+Bev						
0.08 (0.00- 1.65)	0.19 (0.02- 1.87)	0.37 (0.15- 0.93)	0.38 (0.06- 2.40)	0.43 (0.06- 3.12)	0.49 (0.14- 1.77)	0.56 (0.31- 1.01)	0.58 (0.43- 0.76)	0.60 (0.40- 0.91)	CT(A)+Pembro					
0.07 (0.00- 1.68)	0.17 (0.01- 1.97)	0.33 (0.09- 1.18)	0.34 (0.04- 2.62)	0.39 (0.04- 3.37)	0.44 (0.09- 2.08)	0.50 (0.17- 1.44)	0.52 (0.21- 1.29)	0.54 (0.21- 1.42)	0.90 (0.35- 2.34)	CT				
0.05 (0.00- 2.62)	0.12 (0.00- 3.58)	0.24 (0.02- 3.40)	0.24 (0.01- 5.40)	0.28 (0.01- 6.70)	0.31 (0.02- 5.21)	0.36 (0.03- 4.65)	0.37 (0.03- 4.56)	0.39 (0.03- 4.87)	0.64 (0.05- 8.04)	0.71 (0.07- 7.42)	Mono+Erib			
0.06 (0.00- 1.64)	0.14 (0.01- 1.99)	0.28 (0.06- 1.36)	0.29 (0.03- 2.72)	0.33 (0.03- 3.48)	0.38 (0.06- 2.29)	0.43 (0.11- 1.73)	0.44 (0.12- 1.62)	0.46 (0.12- 1.76)	0.77 (0.20- 2.89)	0.86 (0.18- 4.18)	1.20 (0.07- 20.41)	Com		
0.07 (0.00- 1.45)	0.16 (0.02- 1.65)	0.32 (0.12- 0.86)	0.32 (0.05- 2.13)	0.37 (0.05- 2.77)	0.42 (0.11- 1.60)	0.48 (0.24- 0.96)	0.49 (0.31- 0.78)	0.52 (0.30- 0.90)	0.86 (0.50- 1.47)	0.96 (0.34- 2.66)	1.34 (0.10- 17.35)	1.12 (0.28- 4.40)	CT(A)+Nivo	
0.04 (0.00- 1.53)	0.09 (0.00- 2.03)	0.17 (0.02- 1.80)	0.17 (0.01- 2.99)	0.20 (0.01- 3.74)	0.22 (0.04- 1.35)	0.26 (0.03- 2.43)	0.26 (0.03- 2.36)	0.28 (0.03- 2.53)	0.46 (0.05- 4.17)	0.51 (0.05- 5.47)	0.72 (0.03- 20.17)	0.60 (0.05- 7.58)	0.53 (0.06- 4.99)	CT(A)+Olap

The grey area indicates a statistically significant comparison. Different interventions are represented by the blue areas.

### 3.6 Safety

Adverse events of 15 interventions were reported, which are detailed in **Table 3**.

**Table 3:** Grade 3-5 AEs.

	Anemia	Neutropenia	Febrile neutropenia	Thrombocyt-openia	Nausea and vomiting	Diarrhoea	Mucositis	Skin and subcutaneo-us tissuedise-ases	Neurotoxi-city	Hepatoto-xicity	Cardiac disorde-rs
Com	1.53%	27.69%	NA	NA	4.61%	7.69%	NA	20.00%	3.08%	7.69%	0.00%
Com(A)	6.25%	47.65%	10.94%	7.80%	3.12%	0.78%	0.78%	0.78%	0.00%	1.56%	NA
CT	0.00%	4.59%	11.49%	0.00%	0.00%	0.00%	1.15%	2.29%	3.45%	NA	NA
CT(A)	4.78%	39.27%	1.71%	1.21%	4.64%	3.05%	1.46%	2.80%	1.33%	1.94%	0.28%
CT(A)+Bev	1.26%	66.15%	0.28%	1.47%	1.33%	1.26%	11.00%	3.71%	0.42%	NA	0.14%
CT(A) +Dmab	6.10%	60.74%	NA	6.10%	1.10%	2.65%	NA	NA	3.71%	3.71%	NA
CT(A) +Erib	NA	16.67%	3.30%	NA	0.00%	NA	0.00%	NA	NA	NA	NA
CT(A) +Olap	2.90%	43.47%	NA	0.00%	1.32%	1.45%	0.00%	0.00%	1.45%	1.45%	NA
CT(A)+Pembro	NA	NA	NA	NA	NA	NA	NA	NA	NA	NA	NA
Mono	0.00%	8.77%	5.26%	0.00%	1.75%	1.75%	0.00%	1.75%	1.75%	1.75%	1.75%
Mono +Erib	0.00%	22.22%	5.55%	0.00%	0.00%	0.00%	0.00%	0.00%	0.00%	NA	NA
Letr+Ever	0.00%	20.00%	NA	NA	0.00%	0.00%	0.00%	0.00%	NA	0.00%	NA
Letr+Ribo	0.00%	43.14%	0.00%	0.00%	0.00%	0.00%	0.00%	NA	0.00%	19.61%	NA
Gose+tamo	NA	NA	NA	NA	NA	NA	NA	NA	NA	NA	NA
Letr+Palb	NA	0.00%	0.00%	NA	NA	NA	NA	NA	NA	NA	NA
CT(A)+Nivo	42.36%	18.70%	NA	3.43%	51.90%	29.77%	6.87%	26.33%	11.06%	22.14%	2.67%

The predominant grade 3-5 adverse effects observed were anemia, neutropenia, thrombocytopenia, nausea/vomiting, diarrhea, stomatitis, mucositis, skin and subcutaneous tissue diseases, sensory neuropathy, hepatotoxicity, and heart problems. Patients treated with CT (A) + Bev (66.15%), CT (A) + Dmab(60.74%) had a higher incidence of grade 3-5 neutropenic adverse events. Patients treated with CT(A)+Nivo had the highest incidence of grade 3-5 anaemia(42.36%), nausea and vomiting (51.90%), and diarrhoea (29.77%). Com (20.00%) and CT(A)+Nivo(26.33%) therapeutic measures cause skin and subcutaneous tissue disease more commonly.The remaining governance measures had low rates of grade 3-5 adverse events.However, some studies did not report grade 3-5 adverse events, and we were not able to know the incidence of adverse events resulting from them.

### 3.7 Publication bias

**Figure 8:**
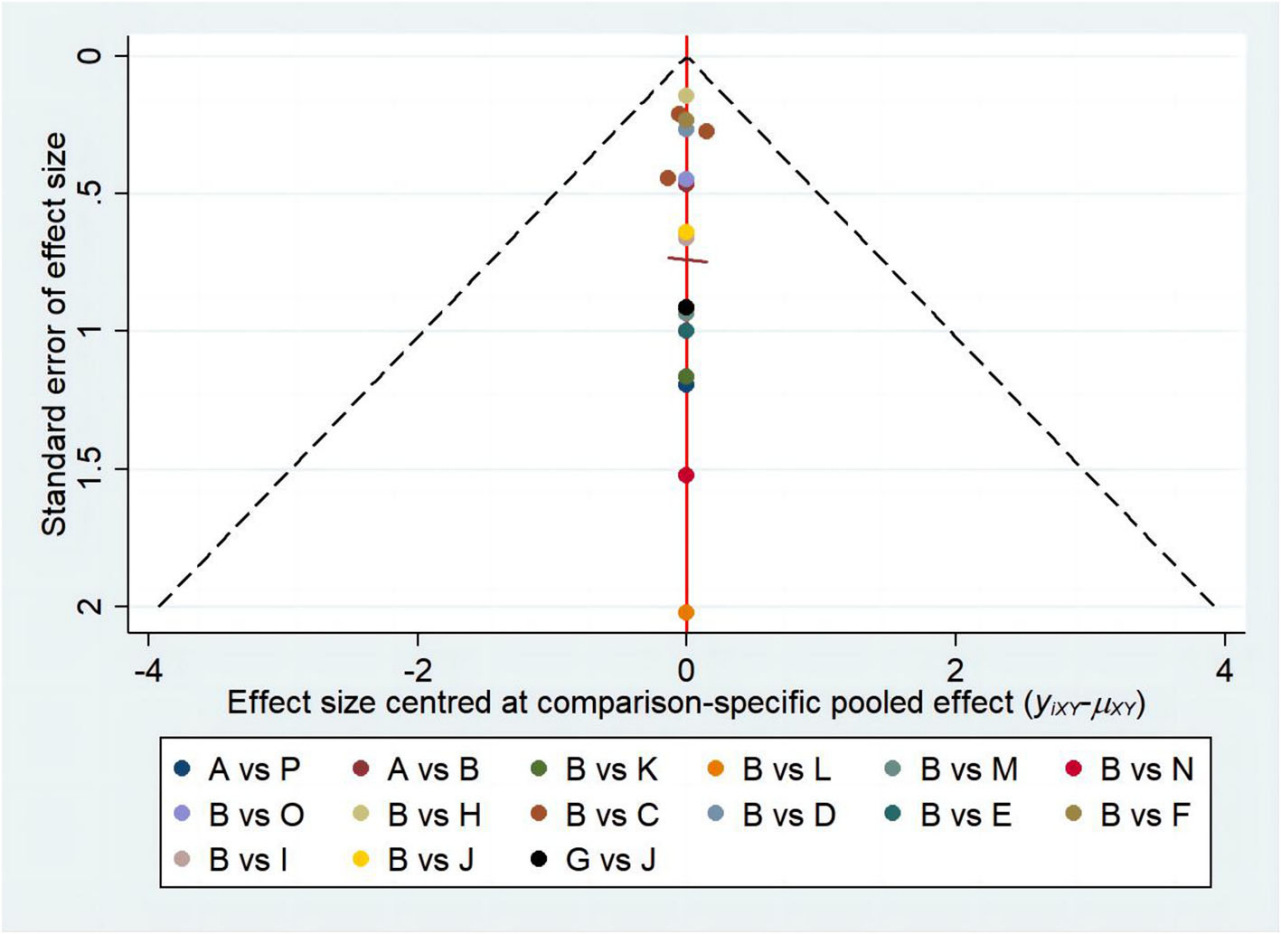
Publication of bias diagrams.

The funnel plot method is the most simple and practical way to judge publication bias, which can make a visual judgement on whether the effect size estimates are related to the sample size, and judge whether there is publication bias by observing whether the distribution of the scatter plot is symmetrical or not. The study included more than 10 papers, so it was appropriate to use a funnel plot for judgement. The comparison-correction funnel plot revealed that the distribution of studies is predominantly centered around the central line, with minimal dispersion on either side. This indicates a minimal probability of publishing bias (**Figure 8**).

## 4 Discussion

The primary outcome of the 17 RCTs included in this analysis was pCR. The purpose of these trials was to assess the effectiveness and safety of various treatment regimens as neoadjuvant therapy in patients with HER2-negative, hormone receptor-positive breast cancer.The 17 RCTs included 16 different intervention arms, which were categorized into 16 treatment regimens based on the study’s design. The present network meta-analysis compared the pCR of the 16 treatment regimens in the reticulated evidence map. The results revealed that the top 5 treatment regimens in terms of pCR were: CT(A)+olaparib group (82.5%), CT(A)+nivolumab (76.5%), Com group (74.9%), CT group (72.1), Moon+eribulin (72.0%).

The general understanding of breast cancer has undergone a significant transformation due to the comprehensive analysis of its molecular characteristics. This analysis now encompasses immunohistochemical markers such as ER, PR, HER2 (ERBB2), and the proliferation marker protein Ki-67 (MKI67), genomic markers including BRCA1, BRCA2, PIK3CA, as well as immune markers like tumour-infiltrating lymphocytes and PD-L1 (32). Neoadjuvant treatment is becoming a prevalent choice for treating early breast cancer in patients with triple-negative and HER2-positive subtypes. The therapeutic regimen consists of endocrine therapy, anti-HER2 targeting, and chemotherapy, tailored to the specific clinical tumor subtype. The use of neoadjuvant chemotherapy (NACT) in HR+/HER2-negative malignancies is a subject of debate (2). HR+/HER2- subtypes of breast cancer are the most common and exhibit significant heterogeneity. Out of the several types, Luminal B type has a higher level of immunogenicity compared to Luminal A type. Similarly, histological grade 3 shows greater immunogenicity than histological grade 1-2. Simultaneously, these cells exhibit increased proliferation, decreased differentiation, poorer prognosis, and necessitate more intense therapeutic interventions. Consequently, it is imperative to evaluate the efficacy of immunotherapy in this particular group (33).

Chemotherapy is a crucial component of neoadjuvant therapy since it aids in the reduction of tumor size, disease management, and the prevention of metastasis. The current chemotherapy regimen for early breast cancer involves the administration of anthracyclines and paclitaxel either in combination or one after the other, spanning a period of 18-24 weeks (2). Frequently employed chemotherapeutic drugs consist of combination regimens such as the AC regimen (doxorubicin + cyclophosphamide) or the TAC regimen (paclitaxel + doxorubicin + cyclophosphamide). Neoadjuvant treatment frequently involves using endocrine therapy as the initial option because HR-positive breast cancer is susceptible to hormones. Widely utilized medications for endocrine therapy include oestrogen receptor modulators like Tamoxifen, as well as non-steroidal aromatase inhibitors like Anastrozole and Letrozole (34). In cases of HER2-negative breast cancer, where anti-HER2 targeted therapy is not applicable, alternative targeted medicines may still be suitable. For instance, the utilization of CDK4/6 inhibitors (such as pembrolizumab) in conjunction with endocrine therapy can enhance therapeutic results.

Nevertheless, our study revealed that breast cancer patients with the HR+/HER2- molecular subtype who underwent neoadjuvant therapy using a treatment regimen consisting of both chemotherapy and immune checkpoint inhibitors therapy did not exhibit any significant statistical variation in pathological complete response (PCR) as compared to patients who only received chemotherapy. KEYNOTE-756 The trial enrolled 1,278 patients with ER+/HER2-, histological grade 3, T1c-T2/cN1-2 or T3-4/cN0-2 breast cancer, with the primary endpoints of pCR (ypT0/Tis, ypN0) and event-free survival (EFS). With a median follow-up of 33.2 months, the pabolizumab combination chemotherapy group had a significantly higher pCR rate compared to the placebo combination chemotherapy group (24.3% vs 15.6%) (35). The CheckMate-7FL study was designed very similarly to the KEYNOTE-756 study to evaluate the efficacy and safety of nabulizumab in combination with NACT and adjuvant endocrine therapy in patients with high-risk ER+/HER2- high-risk breast cancer. A total of 510 patients with ER+/HER2-, histological grade 2 (ER percentage of 1%-10%) or grade 3, T1c-T2/cN0-cN2 or T3-T4/cN0-cN2 were enrolled in the study. The study also met its single primary endpoint of pCR (ypT0/Tis, ypN0), showing an improved pCR rate in the nabulizumab-treated group (24.5% vs 13.8%) (36). These results confirm the findings of the I-SPY2 study (37), which showed that the addition of pembrolizumab or doxorubicin to neoadjuvant chemotherapy in patients with ER+/HER2-, MammaPrint high-risk breast cancers increased the pCR rate (38, 39). Both the CheckMate-7FL study and the KEYNOTE-756 study used histological grade as one of the selection criteria. In these trials, the inclusion of patients with histological grade 3, which is very sensitive to endocrinology, may have weakened the impact of pCR on survival. In the I-SPY2 trial, only ER+/HER2- patients with the highest MammaPrint risk (MP-high 2) benefited from ICI. They were characterised by high immune infiltration, high proliferation and lower sensitivity to endocrine therapy. Although MP-high 2 breast cancers are almost always histological grade 3, less than one third of ER+/HER2- breast cancers with histological grade 3 are MP-high 2 (39). Long-term survival results from the KEYNOTE-756 study will need to continue to be awaited before NACT combined with immunotherapy becomes the standard of care for patients with high-risk ER+/HER2- breast cancer.

Chemotherapy combined with immunotherapy does present some challenges for patients with HR+/HER2- breast cancer. In the KEYNOTE-522 (40) and IMpassion130 (41) trials, chemotherapy combined with immunotherapy resulted in significantly higher pCR rates in patients with triple-negative breast cancer, while the effect was more limited in HR+/HER2- patients. One reason for this lies in the biology of HR+/HER2- breast cancers. This type of cancer usually responds better to hormone therapy but is less sensitive to chemotherapy and immunotherapy. Another reason is differences in the immune microenvironment; the tumour microenvironment in HR+/HER2- breast cancers may be more suppressive of immune cell activity, reducing the effectiveness of immunotherapy.

HR+/HER2- breast cancer is highly sensitive to hormone therapy, a property that makes endocrine therapy highly effective in these patients. Long-term endocrine therapy significantly improves disease-free survival and overall survival, meaning that even if a patient does not achieve a pCR after NAT, significant clinical benefit can still be achieved with continued endocrine therapy (42). This treatment sensitivity further undermines the importance of pCR as the only prognostic indicator (43). Although HR+/HER2- breast cancer is less responsive to neoadjuvant therapy than other breast cancer subtypes and PCR is difficult to obtain (9), it can still benefit from this approach, particularly in terms of improved objective tumour remission and breast conservation rates (44, 45). The assessment of the outcome of HR+/HER2- breast cancer in clinical practice requires a combination of factors that are not solely dependent on pCR. Other important prognostic factors include tumour size, lymph node status, tumour grade and hormone receptor status (42, 46). Together, these factors affect the long-term prognosis of the patient and are essential for the development of an individualised treatment plan. Ignoring these factors and focusing only on pCR may lead to incomplete or biased treatment decisions (47).

Nevertheless, given the limited clinical evidence supporting neoadjuvant endocrine therapy, it is advisable to prioritize chemotherapy as the preoperative treatment for hormone receptor-positive breast cancer. Neoadjuvant endocrine therapy may be considered as an alternative for hormone-dependent patients who are not suitable for chemotherapy or do not respond well to chemotherapy. Research findings indicate that the combination of CDK4/6 inhibitors with neoadjuvant endocrine therapy can greatly enhance the inhibitory impact on tumor cells. Additionally, the rate of complete cell cycle arrest is higher at 36% (48). Therefore, the use of endocrine drugs in conjunction with CDK4/6 inhibitors is a viable alternative for neoadjuvant treatment. Due to the low rate of pathological complete response (pCR) in hormone receptor-positive breast cancer, the selection of adjuvant treatment choices should primarily be based on the patient’s clinical recurrence risk.

Our study’s main advantage was its inclusion of the biggest number of RCTs with sufficient sample sizes, surpassing prior meta-analyses. The protocols were ranked quantitatively and intuitively using NMA. The confluence of efficacy and safety events facilitated a full assessment of each treatment regimen. However, it is important to note that there are some potential limitations in this NMA. To close the loop on the NMA, we excluded some single-arm studies. Specific to individual treatment options, the number of included literature and the sample of patients is relatively insufficient. Furthermore, all computations were derived from publicly available findings rather than individualized data. The final constraint is an intrinsic defect within the NMA itself, which is unavoidable.

In conclusion, the groups receiving CT(A)+Olaparib and CT(A)+nivolumab showed greater effectiveness in neoadjuvant therapy for HR+/HER2- breast cancer. Moreover, it is imperative to prioritise the efficient management of the negative consequences of the treatment regimen in order to improve the patient’s capacity to endure it. In order to confirm the results of this study, it is important to conduct more well-designed and appropriate RCTs within the limitations of the current research.

## Data Availability

The original contributions presented in the study are included in the article/**Supplementary Material**. Further inquiries can be directed to the corresponding authors.
